# Matrix Remodeling Enhances the Differentiation Capacity of Neural Progenitor Cells in 3D Hydrogels

**DOI:** 10.1002/advs.201801716

**Published:** 2019-01-11

**Authors:** Christopher M. Madl, Bauer L. LeSavage, Ruby E. Dewi, Kyle J. Lampe, Sarah C. Heilshorn

**Affiliations:** ^1^ Department of Bioengineering Stanford University Stanford CA 94305 USA; ^2^ Department of Materials Science and Engineering Stanford University Stanford CA 94305 USA; ^3^ Department of Chemical Engineering University of Virginia Charlottesville VA 22904 USA

**Keywords:** hydrogel degradation, matrix remodeling, neural progenitor cells, stem cell differentiation

## Abstract

Neural progenitor cells (NPCs) are a promising cell source to repair damaged nervous tissue. However, expansion of therapeutically relevant numbers of NPCs and their efficient differentiation into desired mature cell types remains a challenge. Material‐based strategies, including culture within 3D hydrogels, have the potential to overcome these current limitations. An ideal material would enable both NPC expansion and subsequent differentiation within a single platform. It has recently been demonstrated that cell‐mediated remodeling of 3D hydrogels is necessary to maintain the stem cell phenotype of NPCs during expansion, but the role of matrix remodeling on NPC differentiation and maturation remains unknown. By culturing NPCs within engineered protein hydrogels susceptible to degradation by NPC‐secreted proteases, it is identified that a critical amount of remodeling is necessary to enable NPC differentiation, even in highly degradable gels. Chemical induction of differentiation after sufficient remodeling time results in differentiation into astrocytes and neurotransmitter‐responsive neurons. Matrix remodeling modulates expression of the transcriptional co‐activator Yes‐associated protein, which drives expression of NPC stemness factors and maintains NPC differentiation capacity, in a cadherin‐dependent manner. Thus, cell‐remodelable hydrogels are an attractive platform to enable expansion of NPCs followed by differentiation of the cells into mature phenotypes for therapeutic use.

## 1 Introduction

Neural progenitor cells (NPCs) are capable of differentiation into the major cell lineages of the central nervous system, including neurons, astrocytes, and oligodendrocytes.^1^ Accordingly, NPCs have garnered substantial interest as potential stem cell therapies to replace damaged nervous tissue in conditions ranging from spinal cord injury to neurodegenerative diseases.^1^ However, despite promising preclinical results, NPC‐based therapies have not yet achieved the clinical success necessary to make regenerative therapies for the central nervous system broadly available to patients.^2^

One major challenge limiting clinical translation is the difficulty and cost associated with expanding the large numbers of NPCs required for use in patients, while retaining the regenerative potential of the cells.^3^ We recently demonstrated that culturing NPCs in 3D hydrogels that permit cell‐mediated matrix remodeling can maintain the stem cell phenotype, or “stemness,” of the NPCs in vitro, and allow for significant NPC expansion in high cell density cultures.^4^ More broadly, hydrogels have shown significant promise as artificial extracellular matrix (ECM) materials for 3D cell culture.^5^ Various synthetic and naturally derived hydrogel materials have been engineered to recapitulate aspects of the native ECM, including stiffness, viscoelasticity, proteolytic degradability, cell adhesivity, and soluble factor presentation, all of which have been shown to modulate stem cell maintenance and differentiation.^6^

While many approaches have focused on transplantation of naïve NPCs to sites of tissue damage, allowing the NPCs to differentiate in vivo into the various cell types necessary for repair, others have sought to use terminally differentiated cells to directly replace the dead or diseased cells responsible for the condition, such as motor neurons in spinal cord injury or dopaminergic neurons in Parkinson's disease.^7^ For neuronal replacement therapies in particular, substantial expansion of the stem cell population is necessary, as differentiated neurons are postmitotic and can no longer divide, meaning that proliferation to achieve the appropriate cell numbers must occur prior to terminal neuronal differentiation.

**Figure 1:**
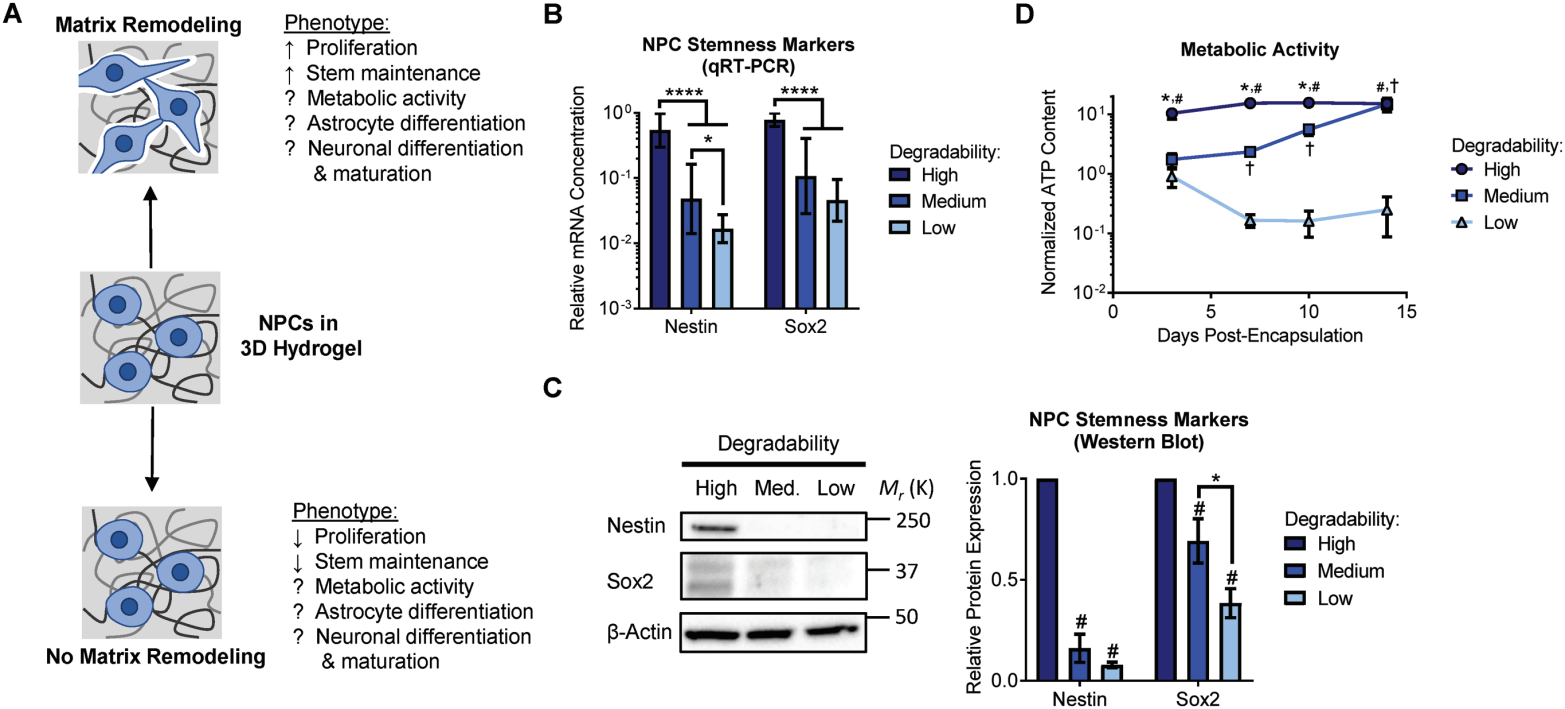
Matrix remodeling modulates NPC stemness and metabolic activity. A) Diagram depicting how matrix remodeling impacts NPC stemness, highlighting that the role of matrix remodeling in NPC metabolic activity, differentiation, and maturation remained unknown. B) Expression of mRNA for the NPC stemness markers nestin and Sox2 decreases with decreasing hydrogel degradability after 7 d in culture. Data are presented as geometric mean with 95% confidence intervals. *p < 0.05, ****p < 0.0001, one‐way ANOVA with Bonferroni post‐hoc test, *n* = 8. C) Western blots for nestin and Sox2 confirm that protein levels of these stemness markers decrease in gels with decreasing degradability after 7 d in culture. Data are mean ± standard error of the mean (s.e.m.), normalized to β‐actin. #*p* < 0.05 compared with a relative expression of 1 (i.e., normalized to the high degradability gels), one sample Student's *t*‐test. *p < 0.05, two‐tailed Student's *t*‐test. *n* = 4 for nestin, *n* = 5 for Sox2. D) Metabolic activity is maintained in high degradability hydrogels, but is significantly decreased in low degradability gels. Over time, the metabolic activity of NPCs in medium degradability hydrogels increases as the cells remodel the gels. Metabolic activity (ATP content) is normalized to DNA content. Data are mean ± s.e.m. *p < 0.05 for high relative to medium degradability, #*p* < 0.05 for high relative to low degradability, and †*p* < 0.05 for medium relative to low degradability at a given time point, two‐way ANOVA with Bonferroni post‐hoc test, *n* = 6–15.

Therefore, beyond simply expanding NPCs in a stem cell state, an ideal material platform would also permit robust differentiation of the cells into a desired mature phenotype. This would decrease processing time and costs associated with using an initial platform to expand the cells, followed by a second platform to differentiate them. Given that cell‐remodelable 3D hydrogels facilitate expansion and stemness maintenance of NPCs, we sought to determine the effect of 3D matrix remodeling on NPC differentiation and maturation (**Figure** **1**A).

Herein, we report that matrix remodeling is required for NPC differentiation into neurons and astrocytes in 3D hydrogels. NPCs cultured within proteolytically degradable hydrogels express lineage‐specific neuronal and astrocytic markers and mature into neurotransmitter‐responsive neurons only if sufficient matrix degradation occurs prior to induction of differentiation. The expression of the transcriptional co‐activator Yes‐associated protein (YAP) by encapsulated NPCs varies with matrix remodeling, and YAP‐mediated signaling is necessary for maintenance of NPC differentiation potential. We propose a mechanism by which matrix remodeling regulates cadherin cell–cell contacts, which in turn modulate β‐catenin signaling to activate YAP expression, thereby priming the NPCs for differentiation.

## 2 Results

### 2.1 NPC Stemness is Regulated by Matrix Remodeling

To interrogate the effects of matrix remodeling on NPC phenotype, NPCs derived from adult murine dentate gyrus were encapsulated within elastin‐like protein (ELP) hydrogels. The modular design of ELPs facilitates simultaneous tuning of several material properties known to impact cell behavior in 3D materials, including the cell‐adhesive ligand concentration, matrix stiffness, and matrix degradability.^4^, ^8^, ^9^ These recombinant protein‐based hydrogels offer several advantages over commonly used synthetic and naturally derived materials. The recombinant design affords precise control over the sequence of the amino acid building blocks, specifying bioactivity, such as cell‐adhesion sites and proteolytic cleavage sequences, and structural properties, such as specific sites to be used in crosslinking.^10^ In contrast, synthetic polymers must be functionalized with bioactive peptides to mimic native ECM biochemistry, and naturally derived materials have limited control over the location of the crosslinking sites within the polymers.

The ELPs used in this study were designed with alternating bioactive domains and structural domains (Figure S1A, Supporting Information). The bioactive domains contain an extended RGD amino acid sequence derived from fibronectin to permit cell adhesion,^8^ as well as an amino acid sequence susceptible to degradation by a disintegrin and metalloprotease 9 (ADAM9), a membrane‐bound protease that we previously reported to be a potent regulator of NPC‐mediated matrix remodeling in ELP matrices.^4^ The structural domains contain the elastin‐like VPGXG amino acid sequence, where the X residue can be any amino acid other than proline. In the ELPs used in this study, one in five of the X amino acids is a lysine (K) to provide primary amines for crosslinking into hydrogels. All hydrogels used in this study were comprised of 3% w/v ELP, as these hydrogels exhibited elastic moduli similar to that of native brain tissue (*E* ≈ 0.5–1.5 kPa) (Table S1, Supporting Information). Furthermore, previous studies in both 2D and 3D hydrogel systems have demonstrated that this stiffness range is capable of supporting differentiation into both neurons and astrocytes,^11^, ^12^ suggesting that differentiation can occur in each stiffness and allowing a focus instead on matrix remodeling. Hydrogel degradability was controlled by varying the crosslink density of the network, resulting in hydrogels with high, medium, and low degradability (Figure S1B and Table S1, Supporting Information).

To assess the effect of matrix remodeling on NPC phenotype, NPCs encapsulated within ELP hydrogels with varying degradability were cultured in stem cell expansion medium for up to 2 weeks. Consistent with our previous results,^4^ the expression of mRNA transcripts of the NPC stemness markers nestin and Sox2 decreased with decreasing hydrogel degradability after 1 week in culture (Figure 1B). We validated these trends on the protein level by Western blot for nestin and Sox2. As expected, both nestin and Sox2 expression were highest in high degradability hydrogels following 1 week of culture (Figure 1C). Importantly, culturing the NPCs in expansion medium did not result in spontaneous differentiation, as measured by expression of mRNA transcripts of several neuron, astrocytic, and oligodendrocyte‐associated genes (Figure S2, Supporting Information).

As NPCs cultured within low degradability hydrogels both lose stemness and do not spontaneously differentiate, we next sought to measure the metabolic activity of the NPCs as an additional metric of cell state. Throughout 2 weeks of culture, NPCs in high degradability gels maintain stable and relatively high levels of adenosine triphosphate (ATP), while NPCs cultured in low degradability gels exhibited a significant and sustained decrease in ATP content (Figure 1D). NPCs encapsulated in hydrogels with medium degradability also initially exhibited decreased ATP content relative to NPCs in high degradability gels. However, as the cells further remodeled the medium degradability hydrogels over time, ATP content increased to levels similar to high degradability hydrogels by day 14 in culture (Figure 1D). The decrease in ATP content measured in low degradability gels appears to correlate with a decreased energetic state, rather than an increase in cell death, as NPCs in all hydrogel conditions remain viable, as indicated by a membrane integrity stain (Figure S3A, Supporting Information). Furthermore, no significant activation of caspase‐3/7, an apoptotic marker, was observed in the low degradability gels relative to the high degradability gels after 7 or 14 d in culture (Figure S3B, Supporting Information).

### 2.2 Matrix Remodeling Is Required for NPC Differentiation

NPCs best maintain their stemness in high degradability hydrogels.^4^ Therefore, a system to both expand and then differentiate NPCs should be highly degradable. To investigate NPC differentiation within 3D hydrogels, NPCs were encapsulated in high degradability ELP hydrogels and cultured in either astrocytic or neuronal differentiation medium. The role of matrix remodeling was evaluated by culturing the NPCs in expansion medium for either 1 d, at which point matrix remodeling has just begun, or 7 d, at which point matrix remodeling is essentially completed, prior to transitioning to the appropriate differentiation medium. Metabolic activity was assessed by measuring the ATP content of the cultures over the course of 2 weeks, and differentiation was assessed by immunocytochemical staining for protein markers of differentiation after 2 weeks of total culture.

**Figure 2:**
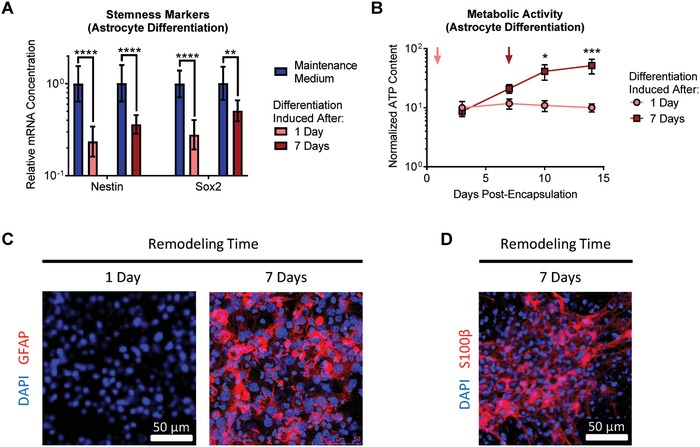
Matrix remodeling enhances NPC differentiation into astrocytes. A) Induction of astrocytic differentiation results in decreased expression of the NPC stemness markers nestin and Sox2, regardless of the duration of matrix remodeling permitted prior to induction of differentiation. Data are presented as geometric mean with 95% confidence intervals. **p < 0.01, ****p < 0.0001, one‐way ANOVA with Bonferroni post‐hoc test, *n* = 8. B) While induction of astrocytic differentiation after 1 d of remodeling does not alter the metabolic activity of the encapsulated cells, waiting until after 7 d of remodeling results in a significant increase in metabolic activity upon induction of differentiation. Data are mean ± s.e.m., normalized to DNA content. *p < 0.05, ***p < 0.001, for differentiation induced at 7 d relative to 1 d at a given time point, two‐way ANOVA with Bonferroni post‐hoc test, *n* = 6–8. C) Only after 7 d of remodeling, followed by induction of astrocytic differentiation for 1 week, do encapsulated cells stain positive for the differentiated astrocytic marker glial fibrillary acidic protein (GFAP). D) Cells provided 7 d of remodeling prior to induction of differentiation also stain positive for the astrocytic marker S‐100 calcium binding protein β (S100β).

To selectively induce astrocytic differentiation, the NPC culture medium was supplemented with bone morphogenetic protein 4 (BMP‐4) in the absence of epidermal growth factor (EGF) and fibroblast growth factor 2 (FGF‐2). When the culture medium was changed after 1 d of matrix remodeling, expression of the stemness markers nestin and Sox2 is significantly decreased after 1 week of culture, as expected (**Figure** **2**A). The ATP content of the cultures remained largely unchanged over the 2 week culture duration (Figure 2B). However, with only 1 d of remodeling, immunostaining for glial fibrillary acidic protein (GFAP) indicated that no astrocytic differentiation was observed (Figure 2C). In contrast, when the NPCs were allowed to remodel the matrix for 7 d prior to induction of differentiation, robust astrocytic differentiation was observed. As expected, nestin and Sox2 expression was decreased after 1 week (Figure 2A). Interestingly, metabolic activity increased significantly after induction of differentiation (Figure 2B). The additional remodeling time prior to differentiation resulted in markedly increased expression of GFAP (Figure 2C), indicating successful astrocytic differentiation. Staining for the s100 calcium binding protein β (S100β) further confirmed the presence of differentiated astrocytes in cultures with differentiation induced after 7 d of remodeling (Figure 2D).

**Figure 3:**
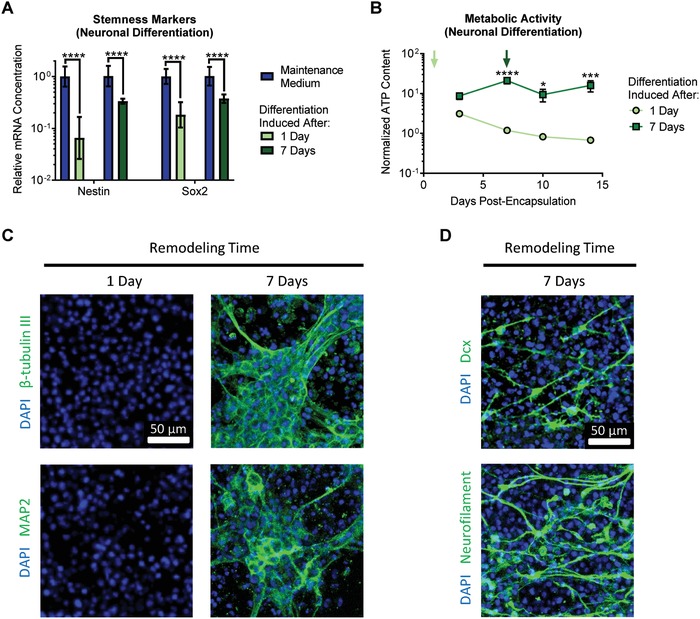
Matrix remodeling enhances NPC differentiation into neurons. A) Induction of neuronal differentiation results in decreased expression of the NPC stemness markers nestin and Sox2, regardless of the duration of matrix remodeling permitted prior to induction of differentiation. Data are presented as geometric mean with 95% confidence intervals. ****p < 0.0001, one‐way ANOVA with Bonferroni post‐hoc test, *n* = 8. B) Induction of neuronal differentiation after 7 d of remodeling results in relatively stable metabolic activity over time, whereas induction of differentiation after only 1 d of remodeling results in significantly decreased metabolic activity. Data are mean ± s.e.m., normalized to DNA content. *p < 0.05, ***p < 0.001, ****p < 0.0001 for differentiation induced at 7 d relative to 1 d at a given time point, two‐way ANOVA with Bonferroni post‐hoc test, *n* = 5–8. C) Only after 7 d of remodeling, followed by induction of neuronal differentiation for 1 week, do encapsulated cells stain positive for the differentiated neuronal markers β‐tubulin III and microtubule‐associated protein 2 (MAP2). D) Cells provided 7 d of remodeling prior to induction of differentiation also stain positive for the early neuronal marker doublecortin (Dcx) and the mature neuronal marker neurofilament.

To induce neuronal differentiation, EGF and FGF‐2 were removed from the NPC culture medium. Similar to the astrocytic differentiation experiments, switching to neuronal differentiation medium resulted in a decrease in expression of the stemness markers nestin and Sox2 (**Figure** **3**A). When neuronal differentiation was induced after only 1 d of matrix remodeling, a significant decrease in ATP content was observed over the 2 week culture duration, whereas ATP content remained consistently higher in cultures provided 7 d of matrix remodeling prior to induction of differentiation (Figure 3B). Consistent with the astrocytic differentiation results, providing only 1 d of matrix remodeling resulted in poor neuronal differentiation, as indicated by negative immunostaining for the neuronal markers β‐tubulin III and microtubule‐associated protein 2 (MAP2) (Figure 3C). In contrast, after 7 d of remodeling, substantial neuronal differentiation was observed, with significant staining for both β‐tubulin III and MAP2 (Figure 3C). Successful neuronal differentiation after 7 d of remodeling was further confirmed by staining for the early neuronal marker doublecortin (Dcx) and the mature neuronal marker neurofilament (Figure 3D).

### 2.3 NPCs Differentiated in Remodelable Matrices Mature into Neurotransmitter‐Responsive Neurons

**Figure 4:**
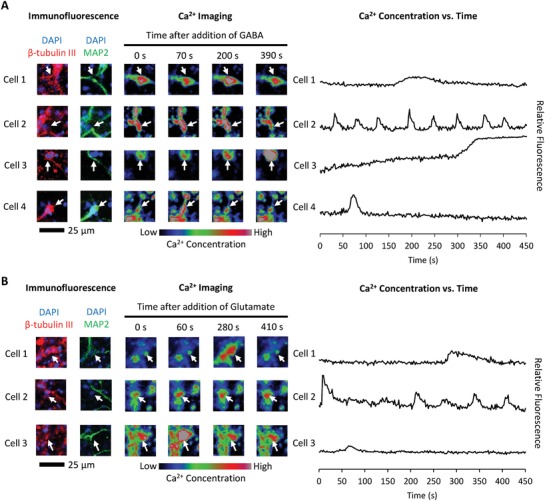
NPCs differentiated in remodelable hydrogels mature into neurotransmitter‐responsive neurons. After 1 week of remodeling followed by 2 weeks of differentiation, encapsulated NPCs were incubated with the calcium‐sensitive Fluo‐4AM dye prior to treatment with either A) γ‐aminobutyric acid (GABA) or B) glutamate. Time lapse confocal microscopy was used to image changes in intracellular calcium concentration. Examples of different types of cellular responses to neurotransmitter treatment are presented. To confirm the imaged cells were differentiated neurons, after calcium imaging, the samples were fixed and immunostained for the differentiated neuronal markers β‐tubulin III and microtubule‐associated protein 2 (MAP2).

While expression of markers such as MAP2 and neurofilament are indicators of mature neuronal differentiation, the true test of neuronal function is the ability to conduct signals via ion currents. NPCs were cultured for 1 week in maintenance medium in high degradability hydrogels, followed by 2 weeks in neuronal differentiation medium to permit neuronal maturation. To assess the electrical activity of neurons within the 3D hydrogels, we stimulated the differentiated NPCs with the neurotransmitters γ‐aminobutyric acid (GABA) and glutamate and observed changes in intracellular calcium concentration by time lapse fluorescence microscopy (**Figure** **4**, and Videos S1 and S2, Supporting Information). Consistent with previous studies employing these techniques in collagen gels,^13^, ^14^ upon neurotransmitter stimulation, the differentiated NPCs exhibited up to four different types of calcium transients: (1) short or (2) long duration increases in intracellular calcium concentration that later return to baseline, (3) sustained increases in intracellular calcium concentration, or (4) a series of transient, consecutive spikes in intracellular calcium concentration. Minimal spontaneous calcium fluxes were observed in the absence of GABA or glutamate treatment (Video S3, Supporting Information). To confirm that the cells responding to neurotransmitter treatment were differentiated neurons, the imaged samples were fixed and immunostained for β‐tubulin III and MAP2. All cells exhibiting changes in calcium concentration triggered by GABA or glutamate stained positive for β‐tubulin III and MAP2 (Figure 4). These results indicate that proteolytically remodelable 3D hydrogels permit neuronal differentiation and maturation.

### 2.4 Matrix Remodeling Promotes YAP‐Mediated Regulation of NPC Phenotype through β‐Catenin Signaling

**Figure 5:**
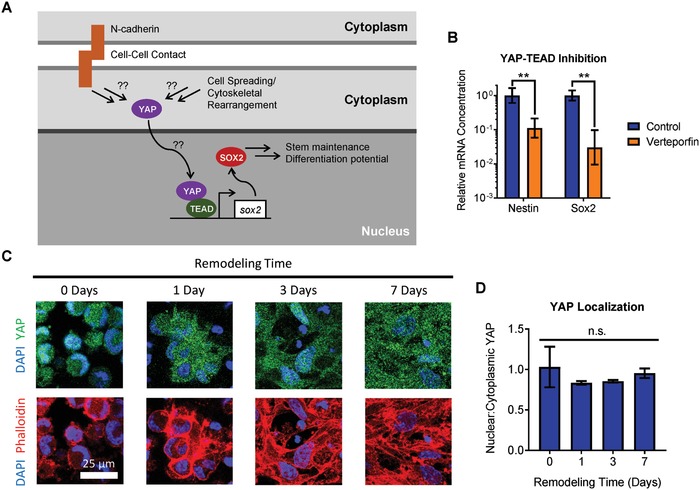
YAP localization does not account for YAP‐dependent effects of matrix remodeling on NPC phenotype. A) Schematic depicting a potential role for matrix remodeling modulating YAP localization through cell spreading and/or cadherin‐mediated cell–cell contact. B) Inhibition of YAP‐TEAD interaction results in decreased expression of the NPC stemness markers nestin and Sox2. Data are presented as geometric mean with 95% confidence intervals. **p < 0.01, two‐tailed Student's *t*‐test, *n* = 4. C) Immunostaining reveals increased cell spreading over time as the NPCs remodel the matrix, but no substantial changes in YAP localization are observed. D) Quantification of YAP localization reveals no significant change in the ratio of nuclear:cytoplasmic YAP as a function of remodeling time. Data are mean ± s.e.m., n.s. = not significant (*p* > 0.05), one‐way ANOVA with Bonferroni post‐hoc test, *n* = 3.

To address the mechanism by which increased matrix remodeling enhances the differentiation capacity of NPCs cultured in degradable 3D hydrogels, we considered the changes in cellular morphology permitted by matrix remodeling. As NPCs remodel the surrounding material, they are free to spread, whereas NPCs in nondegradable hydrogels remain rounded.^4^ Rearrangement of the actin cytoskeleton during cell spreading is known to modulate changes in cellular signaling pathways, notably by regulating the cytoplasmic versus nuclear localization of the transcriptional co‐activator YAP.^15^ Increased spreading is most often correlated with increased nuclear localization of YAP and subsequent activation of YAP responsive gene transcription.^15^, ^16^ Furthermore, other studies have revealed that cadherin‐mediated cell–cell contact can also regulate YAP signaling,^17^ and matrix remodeling can serve to regulate cell–cell contacts. Thus, we postulated that increased cell spreading permitted by increased matrix remodeling could activate YAP signaling through rearrangement of the actin cytoskeleton and/or through maintenance of cadherin cell–cell contacts (**Figure** **5**A). Previous studies have demonstrated that active YAP mediates stemness maintenance in various stem cells, including pluripotent stem cells^18^, ^19^ and intestinal stem cells.^20^ In the case of pluripotent stem cells, YAP was found to be associated with the promoter for the stemness factor Sox2, which is also a key stemness factor for NPCs (Figure 5A).^18^

To determine if active YAP signaling was required for maintenance of NPC stemness, and thus maintenance of differentiation capacity, NPCs cultured in remodelable hydrogels were treated with the small molecule inhibitor verteporfin, which blocks the interaction of YAP with TEA domain (TEAD)‐family transcription factors. Blocking YAP‐TEAD interaction in high degradability hydrogels significantly decreased the expression of nestin and Sox2 compared to control hydrogels, indicating a loss of NPC stemness (Figure 5B). This finding is consistent with the hypothesis that active YAP signaling is required to maintain NPC stemness. We next investigated the role of matrix remodeling on cell spreading and subcellular localization of YAP. Consistent with our previous results, as NPCs remodel the hydrogels, cell spreading increases, as observed by staining for F‐actin (Figure 5C). However, immunocytochemical staining for YAP revealed no significant differences in YAP localization as a function of remodeling time, despite the substantial changes in cellular morphology (Figure 5C,D). YAP activity and subcellular organization is regulated by phosphorylation of specific serine residues in the protein. Phosphorylation at ser127 promotes cytoplasmic localization of YAP, while phosphorylation at ser381 primes YAP for degradation.^21^ Thus, both phosphorylation events are associated with decreased YAP signaling. To validate our immunostaining results, Western blot analysis of YAP phosphorylation was performed. Increased remodeling time did not result in significant changes in YAP phosphorylation (Figure S4, Supporting Information), consistent with the observation that YAP localization did not vary with remodeling time. Furthermore, the subcellular localization of YAP was not correlated with hydrogel degradation in any hydrogel degradability at all time points assayed (Spearman correlation, *p* = 0.58; Figure S5, Supporting Information). Taken together, these results indicate that while YAP signaling is necessary to maintain NPC stemness, matrix remodeling does not regulate YAP activity by modulating YAP localization.

**Figure 6:**
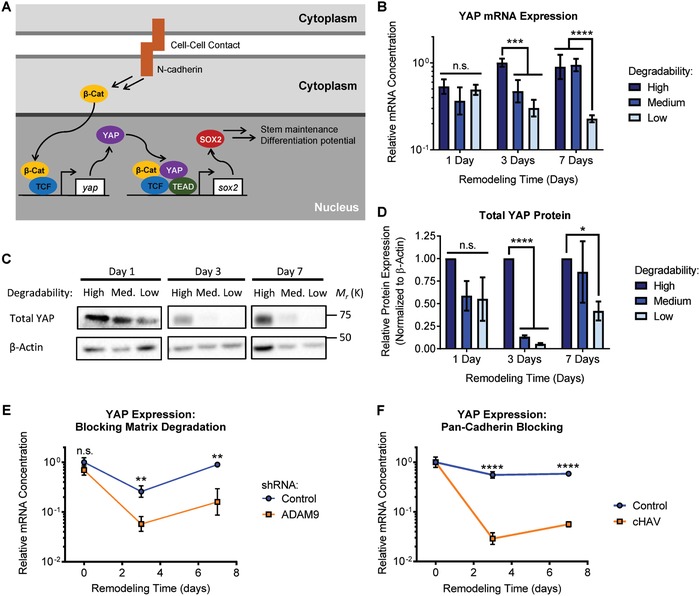
Matrix remodeling regulates YAP expression via cadherin‐mediated cell–cell contact. A) Schematic depicting a mechanism by which increased cadherin‐mediated cell–cell contact facilitated by matrix remodeling regulates YAP expression and NPC phenotype. B) Expression of YAP mRNA varies with remodeling time and hydrogel degradability. Data are presented as geometric mean with 95% confidence intervals. ***p < 0.001, ****p < 0.0001, two‐way ANOVA with Bonferroni post‐hoc test, *n* = 4. C) Western blot analysis confirms decreased YAP protein levels with decreasing hydrogel degradability. D) Western blot quantification is consistent with YAP mRNA measurements, indicating significant variation in YAP protein levels with remodeling time and hydrogel degradability. Data are mean ± s.e.m., normalized to β‐actin. *p < 0.05, ****p < 0.001 compared with a relative expression of 1 (i.e., normalized to the high degradability gels), one sample Student's *t*‐test, *n* = 4. E) Blocking hydrogel degradation by shRNA‐mediated knockdown of the protease ADAM9 or F) inhibiting cadherin‐mediated cell–cell contact results in decreased expression of YAP mRNA. In (E) and (F), data are presented as geometric mean with 95% confidence intervals. n.s. = not significant (*p* > 0.05), **p < 0.01, ***p < 0.001, ****p < 0.0001, two‐tailed Student's *t*‐test, *n* = 3–4.

In addition to subcellular localization, the overall expression levels of YAP can regulate YAP‐associated signaling. We previously reported that matrix remodeling regulates β‐catenin signaling by modulating cadherin cell–cell contacts.^4^ Other studies have demonstrated that β‐catenin signaling can regulate expression of YAP,^22^ and that both β‐catenin and YAP signaling play a role in regulating Sox2 transcription.^18^, ^23^ We thus hypothesized that matrix remodeling could alter YAP signaling by modulating β‐catenin signaling (**Figure** **6**A). Temporal changes in YAP mRNA and protein expression levels are consistent with this hypothesis (Figure 6B–D). Upon encapsulation in a hydrogel, NPCs lose cell–cell contact, and thus exhibit decreased β‐catenin activity.^4^ We anticipated that this loss in β‐catenin signaling would lead to decreased YAP transcription over time if remodeling was not permitted. Accordingly, 1 d postencapsulation, there is a decrease below baseline of YAP mRNA expression in all hydrogels, regardless of degradability (Figure 6B). However, by 3 d of culture, sufficient remodeling had occurred in high degradability gels to restore expression of YAP, while YAP expression was significantly suppressed in medium and low degradability gels (Figure 6B). After 7 d of culture, both high and medium degradability gels exhibited normal levels of YAP expression, whereas expression was still decreased in low degradability gels (Figure 6B). Analysis of YAP protein levels by Western blot confirmed these trends (Figure 6C). No significant difference in total YAP content was observed after 1 d of culture, whereas YAP protein levels were significantly higher in high degradability gels by day 3 (Figure 6D). After 7 d of culture, only low degradability gels still exhibited decreased YAP protein levels (Figure 6D).

To validate that matrix remodeling regulates YAP expression, NPCs that were lentivirally transduced to express an shRNA targeting the protease ADAM9 were encapsulated within high degradability hydrogels. Knockdown of ADAM9 was previously shown to block NPC‐mediated degradation of ELP hydrogels.^4^ Inhibiting matrix remodeling through ADAM9 knockdown resulted in a significant decrease in YAP expression compared to cells transduced with a nonsilencing control shRNA (Figure 6E), confirming that matrix remodeling is required for maintenance of YAP expression. Finally, to confirm that matrix remodeling exerts control over YAP expression via cadherin cell–cell contacts, NPCs encapsulated in high degradability hydrogels were treated with the cyclic histidine‐alanine‐valine (cHAV) peptide to block cadherin binding. As expected, cadherin blocking resulted in decreased YAP expression when compared to control cells (Figure 6F). Together, these data support the hypothesis that cadherin‐mediated contact facilitated by matrix remodeling regulates YAP expression, which in turn is involved in the regulation of NPC stemness maintenance through Sox2 expression, ultimately impacting the differentiation capacity of encapsulated NPCs.

## 3 Discussion

Given the therapeutic potential of NPCs to replace damaged nervous tissue, various strategies have been employed to control material‐induced differentiation of NPCs. Matrix mechanics,^11^, ^24^, ^25^ substrate topography,^26^ cell‐adhesive ligand presentation,^27^ and soluble factor delivery^28^ have all been demonstrated as powerful approaches to modulate the differentiation of NPCs. We recently reported that matrix remodeling was required for NPCs to maintain their stem potential when cultured in 3D hydrogels.^4^ However, our previous work did not directly assess the impact of matrix remodeling on differentiation and neuronal maturation. Here, we identify that matrix remodeling has a dramatic effect on NPC differentiation within 3D hydrogels, with differentiation occurring only after sufficient remodeling has occurred. Importantly, matrix remodeling influences NPC differentiation in a manner distinct from other material‐directed differentiation approaches, in that remodeling is necessary to prime the cells for differentiation, while other cues such as soluble factors (as used in this study) or matrix mechanics^11^, ^24^, ^25^ may be used to bias the fate of the differentiated cells (e.g., neuronal versus astrocytic differentiation).

We previously reported that NPCs cultured within low degradability gels rapidly lost expression of stem cell markers and ceased proliferating (Figure 1A).^4^ One might assume that this result means that low degradability gels promote NPC differentiation, even under maintenance culture conditions. However, this is not the case, as expression of differentiation markers is not increased simply by culturing in low degradability gels (Figure S2, Supporting Information). In contrast, premature induction of differentiation before sufficient matrix remodeling occurred resulted in a diminished capacity to differentiate into either neurons or astrocytes (Figures 2 and 3). NPCs cultured in low degradability gels enter a state with low metabolic activity, neither maintaining their stem cell phenotype nor being primed for differentiation (Figure 1D). However, if sufficient matrix remodeling is permitted, NPCs robustly differentiated into astrocytes (Figure 2) and mature neurons that respond to neurotransmitter stimulation (Figures 3 and 4). These results suggest that matrix remodeling is an important parameter not just when designing materials for NPC expansion, but also for designing materials to enable NPC differentiation.

The transcriptional co‐activator YAP has been implicated as a signal transduction mechanism in material‐induced differentiation of other stem cell types. YAP activity can be modulated either by altering its subcellular localization in the nucleus versus the cytoplasm or by changing the level of YAP protein expressed.^29^ In a biomaterials context, the most commonly studied role of YAP is as a mechanosensor that translocates to the nucleus when cells are cultured on stiff substrates.^15^, ^30^ YAP localization has previously been shown to modulate neuronal differentiation of embryonic stem cells (ESCs), with relatively soft substrates favoring neuronal differentiation due to cytoplasmic localization of YAP.^31^, ^32^ Conversely, nuclear localization of YAP can promote stem cell maintenance in the case of ESCs^32^, ^33^ and intestinal stem cells.^20^ Here, we found that YAP localization was not correlated with changes in NPC phenotype resulting from differing levels of matrix remodeling (Figure 5).

Changes in YAP activity resulting from altered expression of YAP are less studied.^29^ Nevertheless, developmental biology studies have revealed that YAP expression is necessary for maintenance of the neural stem cell population during development.^34^ Furthermore, YAP expression was correlated with an NPC phenotype in cells derived from induced pluripotent stem cells.^35^ These studies are consistent with our findings that YAP activity is required to maintain NPC stem potential and that YAP activity in NPCs is transcriptionally regulated (Figures 5B and 6).

Recently, YAP expression was shown to be mechanosensitive in 2D cultures of primary NPCs.^25^ Similar to the impact of matrix remodeling on YAP in our 3D cultures, YAP localization in this previous study was not affected by matrix mechanics, and the mechanism of YAP‐mediated action implicated crosstalk with β‐catenin signaling.^25^ However, Rammensee et al. found that YAP antagonized β‐catenin signaling by binding and sequestering β‐catenin, thereby inhibiting its proneurogenic function.^25^ Importantly, this mechanism of action does not require YAP‐TEAD interaction. In contrast, inhibition of YAP‐TEAD interaction in our system results in a loss of NPC stemness (Figure 5B). We have previously shown that matrix remodeling activates β‐catenin signaling through cadherin‐mediated cell–cell contact to maintain stemness.^4^ Our matrix degradation inhibition and cadherin blocking studies suggest that β‐catenin signaling plays a role in regulating YAP expression (Figure 6E,F), and that decreased YAP levels due to prolonged loss of β‐catenin signaling can decrease expression of Sox2, a key transcription factor regulating NPC stem potential and differentiation (Figure 6A). The role of β‐catenin in regulating NPC self‐renewal versus differentiation is context dependent, for instance, varying with growth factor signaling.^36^ Future studies should explore the effects of material properties such as 2D versus 3D dimensionality to elucidate possible mechanisms underlying these two different roles for β‐catenin and YAP signaling in the context of biomaterial culture platforms for NPCs.

## 4 Conclusion

Matrix remodeling is required both to maintain NPC stemness and to enable NPC differentiation and maturation within 3D hydrogels. Highly degradable hydrogels facilitate sustained expression of NPC stemness markers and maintain NPC metabolic activity. Even within high degradability gels, matrix remodeling must first occur in order to prime the NPCs for differentiation. When sufficient remodeling time is provided, encapsulated NPCs differentiate into astrocytes and mature neurons. YAP‐TEAD interaction is necessary to maintain expression of NPC stemness factors that enable differentiation. Matrix remodeling regulates YAP activity not by modulating subcellular YAP localization but rather by controlling YAP expression. Blocking matrix degradation or remodeling‐mediated cadherin contact results in a loss of YAP expression, in turn suppressing NPC stemness and differentiation. Culturing NPCs in remodelable hydrogels is thus a powerful strategy to harness β‐catenin and YAP signaling to expand and differentiate neural stem cells.

## 5 Experimental Section

*Materials*: All materials were purchased from either Sigma‐Aldrich or Fisher and used as received, unless otherwise noted.

*Expression and Purification of ELP*: The ELP construct was cloned into pET15b plasmids using traditional molecular biology techniques, as previously described.^8^ Plasmids encoding the ELP were transformed into BL21(DE3)pLysS *Escherichia coli* following the manufacturer's protocol (Life Technologies). ELPs were expressed under control of the T7 promoter by induction with isopropyl β‐D‐1‐thiogalactopyranoside and purified by inverse temperature cycling, as described in detail elsewhere.^37^, ^38^ Protein purity and identity were confirmed by sodium dodecyl sulfate‐polyacrylamide gel electrophoresis (SDS‐PAGE) and Western blot.

*Hydrogel Fabrication and Characterization*: ELP hydrogels with varying degradability were prepared by crosslinking ELPs with the amine‐reactive crosslinker tetrakis(hydroxymethyl)phosphonium chloride (THPC) at varying stoichiometric ratios.^4^ The polymer content of all hydrogels was kept constant at 3% w/v, as hydrogels with this formulation do not exhibit significant changes in swelling, microstructure, or macromolecular transport as a function of crosslinking density.^4^ Hydrogel mechanical properties were characterized by measuring the elastic modulus of hydrated hydrogel samples in unconfined compression using an ARG2 rheometer.^4^

To prepare fluorescently labeled hydrogels to monitor NPC‐mediated matrix degradation, ELPs were dissolved in anhydrous dimethylsulfoxide (DMSO) at 20 mg mL^−1^. To the stirring ELP solution, Cy5‐*N*‐hydroxysuccinimidyl ester (Cy5‐NHS, Lumiprobe) and excess triethylamine were added. Sufficient Cy5‐NHS was added to label one lysine residue per ELP polymer. After 24 h at room temperature, the reaction mixture was diluted in Milli‐Q water and dialyzed against Milli‐Q water to remove DMSO and any unreacted dye. The dialyzed solution was frozen and lyophilized to afford the labeled ELP as a blue solid. Hydrogel degradation was monitored by encapsulating NPCs in hydrogels comprised of a 1:1 mixture of labeled and unlabeled ELP proteins and measuring the fluorescence intensity of the culture medium over time. To convert fluorescence intensity to fraction degraded, the portion of gel remaining after 14 d in culture was fully degraded by treating with 2.5% trypsin (Gibco) to determine the total possible fluorescence signal. Serial dilutions were performed to ensure a linear dynamic concentration range.

*Culture and Encapsulation of NPCs*: Adult murine hippocampal NPCs, isolated from microdissected dentate gyrus,^39^ were kindly provided by Prof. Theo Palmer (Stanford Neurosurgery). NPCs were expanded on polyornithine and laminin‐coated tissue culture plastic in maintenance medium consisting of Neurobasal‐A, 2% B27 supplement, GlutaMAX (Gibco), 20 ng mL^−1^ FGF‐2, and 20 ng mL^−1^ EGF (PeproTech). For encapsulation in ELP hydrogels, NPCs were trypsinized, washed, and counted. The cell pellet was resuspended in a 3.75% w/v solution of ELP in Dulbecco's phosphate‐buffered saline (PBS) at a sufficient concentration to afford a final cell density in the hydrogels of 5 × 10^7^ mL^−1^. THPC diluted in PBS to an appropriate concentration to achieve a 0.5:1, 0.75:1, or 1:1 stoichiometric ratio of THPC:ELP reactive groups was mixed in a 1:4 volumetric ratio with the cell suspension in ELP to yield a final ELP concentration of 3% w/v. Hydrogels were allowed to crosslink at room temperature for 15 min, followed by an additional 15 min at 37 °C prior to addition of cell culture medium. NPCs were maintained in a humidified cell culture incubator at 37 °C with 5% CO_2_, and cell culture medium was replaced every 2 d.

To induce astrocytic differentiation, the culture medium was replaced with Neurobasal‐A, 2% B27 supplement, GlutaMAX, and 10 ng mL^−1^ BMP‐4 (PeproTech). To induce neuronal differentiation, the culture medium was first replaced with Neurobasal‐A, 2% B27 supplement, GlutaMAX, and 5 ng mL^−1^ FGF‐2. After 24 h, the medium was subsequently changed to Neurobasal‐A, 2% B27 supplement, and GlutaMAX. For YAP‐TEAD inhibition studies, NPCs in high degradability gels were cultured with 4 × 10^−6^ m verteporfin. For cadherin inhibition studies, NPCs were treated with 1 mg mL^−1^ cHAV peptide (Exherin, AdooQ Bioscience) prior to encapsulation in high degradability gels and were then cultured with 1 mg mL^−1^ cHAV for 7 d. Medium with fresh inhibitors was replaced every 2 d. To block hydrogel degradation, NPCs that constitutively express an shRNA targeting ADAM9 or a control, nonsilencing shRNA were generated as described previously.^4^ To maintain shRNA expression, these cells were cultured in maintenance medium plus 0.6 µg mL^−1^ puromycin, with medium replaced every 2 d.

*Biochemical Analysis of NPC Phenotype*: For metabolic activity and caspase‐3/7 activation assays, NPC‐containing hydrogels were removed from their silicone molds, transferred to lysis buffer (20 × 10^−3^ m Tris HCl, 150 × 10^−3^ m NaCl, 0.5% Triton X‐100, pH 7.4), and disrupted by sonication. ATP content was determined by assaying lysates with the CellTiter Glo Luminescent Cell Viability Assay (Promega) and relating lysate luminescence to ATP content based on a standard curve. Caspase‐3/7 activity was determined by assaying lysates with the Apo‐ONE Homogeneous Caspase‐3/7 Assay (Promega) and relating lysate fluorescence to caspase activity based on a standard curve prepared from recombinant human caspase‐3 (Millipore). DNA content was determined using the Quant‐iT PicoGreen dsDNA Assay Kit (Life Technologies), following the manufacturer's instructions. ATP content and caspase activity were normalized to DNA content to provide a metric of per cell metabolic activity and apoptotic activity, respectively. Cell viability was also assessed by Live/Dead staining (Life Technologies), imaged on a Leica SPE confocal microscope.

mRNA expression was measured by quantitative reverse transcription‐polymerase chain reaction (qRT‐PCR), following a previously published procedure.^4^ Briefly, the hydrogels were homogenized in TRIzol reagent (Life Technologies), and RNA was isolated by phenol‐chloroform extraction using Phase Lock gels (5 Prime). An amount of 1 µg of RNA per sample was reverse transcribed using a High‐Capacity cDNA Reverse Transcription Kit (Applied Biosystems). PCR was performed on 1 µg of cDNA per sample per gene target using Fast SYBR Green Master Mix (Applied Biosystems) on an Applied Biosystems StepOnePlus Real Time PCR System. Primers are listed in Table S2 (Supporting Information).

Western blots were performed according to a previously published procedure.^4^ Briefly, hydrogels were homogenized in radioimmunoprecipitation assay buffer containing protease and phosphatase inhibitors. The extracted protein was separated by SDS‐PAGE, transferred to a polyvinylidene difluoride membrane, blocked with nonfat milk, and probed with primary antibodies (see Table S3 in the Supporting Information) against the desired protein target. The primary antibodies were detected using horseradish peroxidase‐conjugated secondary antibodies (donkey anti‐mouse or donkey anti‐rabbit, Jackson ImmunoResearch, 1:10000). Blots were developed with SuperSignal West Pico or Femto Chemiluminescent Substrates (Pierce) and imaged using a ChemiDoc MP gel imaging system (Bio‐Rad). Densitometry analysis was performed using ImageJ (NIH), and protein expression was normalized to expression of a loading control.

Immunocytochemical staining was performed as previously described.^4^, ^38^ Briefly, samples were fixed with 4% paraformaldehyde, permeabilized with Triton X‐100, and blocked with a mixture of bovine serum albumin and either goat or donkey serum, depending on the species of the secondary antibody used. Samples were incubated with primary antibodies (see Table S3 in the Supporting Information) overnight at 4 °C and washed. Samples were subsequently stained with fluorescently labeled secondary antibodies (AF488 goat anti‐mouse, AF546 goat anti‐rabbit, AF488 donkey anti‐goat (Life Technologies, 1:500), or AF647 goat anti‐chicken (Abcam, 1:400)) and 4′,6‐diamidino‐2‐phenylindole dihydrochloride (DAPI) as a nuclear counterstain overnight at 4 °C. Samples were washed and mounted for imaging on a Leica SPE confocal microscope.

*Calcium Imaging of Differentiated Neurons*: Time‐lapse imaging of calcium concentrations in differentiated neurons was adapted from a previously published procedure.^13^ After 21 d of culture, NPCs encapsulated in high degradability hydrogels were incubated with 2 × 10^−6^ m Fluo‐4 AM dye (Life Technologies) in Neurobasal‐A medium without phenol red for 1 h at 37 °C. The hydrogel constructs were then washed with normal physiological medium (145 × 10^−3^ m NaCl, 5 × 10^−3^ m KCl, 1.8 × 10^−3^ m CaCl_2_, 0.8 × 10^−3^ m MgCl_2_, 10 × 10^−3^ m 4‐(2‐hydroxyethyl)‐1‐piperazineethanesulfonic acid (HEPES), 10 × 10^−3^ m glucose, pH 7.4). The cells were subsequently maintained in normal physiological medium at room temperature (≈22 °C) for the duration of the experiment. The Fluo‐4 AM stained cells were imaged on a Leica SPE confocal microscope, exciting at 488 nm every 2 s and recording fluorescence emission from a single *z*‐plane (≈2.5 µm thick) for up to 10 min. Baseline images were recorded without addition of neurotransmitters to rule out spontaneous depolarization. To measure cellular responses to neurotransmitter treatment, the cells were exposed to 10 × 10^−6^ m GABA or 50 × 10^−6^ m glutamate and imaged immediately. To quantify changes in relative Fluo‐4 AM fluorescence, corresponding to changes in intracellular calcium concentration, regions of interest were drawn around cells using ImageJ (NIH) and the integrated pixel intensity was recorded at each time point. After live calcium imaging was completed, the samples were fixed with paraformaldehyde and stained for the neuronal markers β‐tubulin III and MAP2, as described above, to ensure neurotransmitter‐responsive cells were differentiated neurons.

*Statistical Analysis*: Two‐tailed Student's *t*‐tests were used for comparisons between two experimental groups. One‐way analysis of variance (ANOVA) with Bonferroni post‐hoc testing was used for comparisons among more than two experimental groups with a single varying parameter. Two‐way ANOVA with Bonferroni post‐hoc testing was used for comparisons among more than two experimental groups with two varying parameters. *P* values of less than 0.05 were considered statistically significant. Independent biological replicates were used to determine *n* values. For western blots, the pixel intensity of all bands was normalized to the high degradability samples to permit comparisons across multiple blots in replicate experiments. Accordingly, to determine statistical significance, one sample Student's *t*‐tests were performed with the null hypothesis that normalized protein expression was equal to 1 (i.e., the expression in the high degradability hydrogels). For analysis of qRT‐PCR data, statistical analysis was performed prior to transforming to a natural scale, as the Δ*C*_T_ values approximate a normal distribution. Relative mRNA expression was therefore reported as a geometric mean with asymmetric 95% confidence intervals derived from the nontransformed data.^40^ Significance was assigned only to changes in mRNA expression greater than twofold. For representative immunostaining images, experiments were repeated at least twice. All statistical analyses were performed using GraphPad Prism 6 software.

## Conflict of Interest

The authors declare no conflict of interest.

## Supporting information

Supplementary

Supplementary

Supplementary

Supplementary
