# Supplementary material for: Matrix Remodeling Enhances the Differentiation Capacity of Neural Progenitor Cells in 3D Hydrogels
Source: Adv Sci (Weinh). 2019 Jan 11;6(4):1801716. doi: 10.1002/advs.201801716 (PMC6382308; doi:10.1002/advs.201801716)
Supplement: Supplementary file 1 — Supplementary [file ADVS-6-1801716-s002.pdf]

Copyright WILEY-VCH Verlag GmbH & Co. KGaA, 69469 Weinheim, Germany, 2019.

ADVANCED SCIENCE
Open Access

Supporting Information

for *Adv. Sci.,* DOI: 10.1002/advs.201801716

Matrix Remodeling Enhances the Differentiation Capacity of Neural Progenitor Cells in 3D Hydrogels

*Christopher M. Madl, Bauer L. LeSavage, Ruby E. Dewi, Kyle J. Lampe, and Sarah C. Heilshorn**

Copyright WILEY-VCH Verlag GmbH & Co. KGaA, 69469 Weinheim, Germany, 2019.

Supporting Information

Matrix remodeling enhances the differentiation capacity of neural progenitor cells in 3D hydrogels

*Christopher M. Madl, Bauer L. LeSavage, Ruby E. Dewi, Kyle J. Lampe, and Sarah C. Heilshorn**

Supplemental Figures……………………………………………………………………2

Supplemental Video Legends…………………………………………………………7

Supplemental Tables……………………………………………………………………8

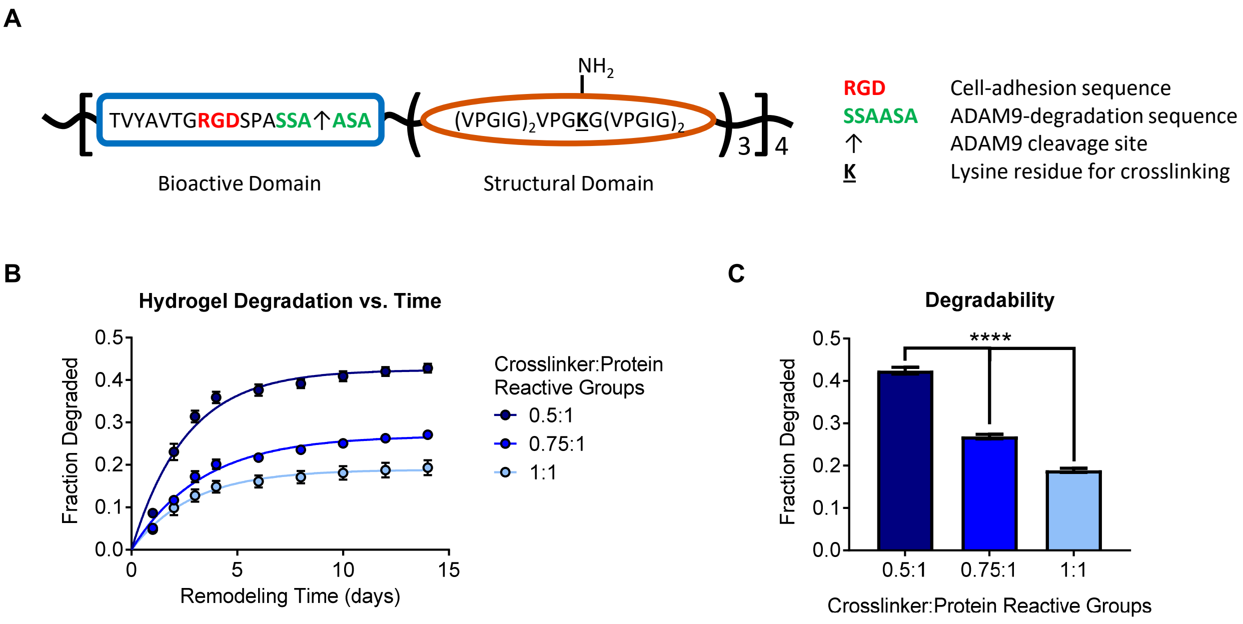

Supplemental Figure S1. Protein engineered hydrogels enable tuning of matrix degradability. **(A)** Schematic depicting the design of the elastin-like proteins (ELPs) used in this study. The bioactive domains of the ELPs include cell-adhesive RGD sequences and sequences susceptible to cleavage by the NPC protease ADAM9. The structural domain contains regularly spaced lysine residues to provide primary amines for crosslinking the proteins into hydrogel networks. **(B)** Varying the stoichiometric ratio of crosslinker to protein reactive groups enables tuning of hydrogel degradability. NPC-mediated degradation of fluorescently-labeled ELPs was measured by monitoring the release of fluorescent material into the cell culture medium over time. Data are mean ± s.d. n=3. **(C)** Maximal hydrogel degradability was quantified by fitting a first-order exponential association model to the hydrogel degradation vs. time data in B. Data are plateau values ± s.e. ****$p<0.0001$, extra sum-of-squares F test, n=3.

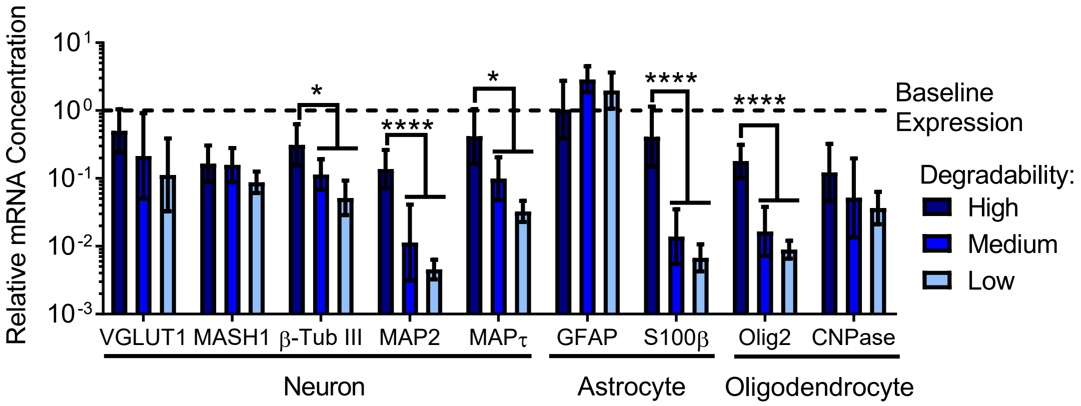

Supplemental Figure S2. NPCs do not spontaneously differentiate under maintenance conditions within 3D ELP hydrogels. Expression of mRNA for various neuron, astrocyte, and oligodendrocyte markers after 3 days of encapsulation does not vary with hydrogel degradability in maintenance medium. Rather, if a significant change occurs, expression of these markers decreases with decreasing degradability. Data are presented as geometric mean with 95% confidence intervals. *$p<0.05$, ****$p<0.0001$, one-way ANOVA with Bonferroni post-hoc test, n=8.

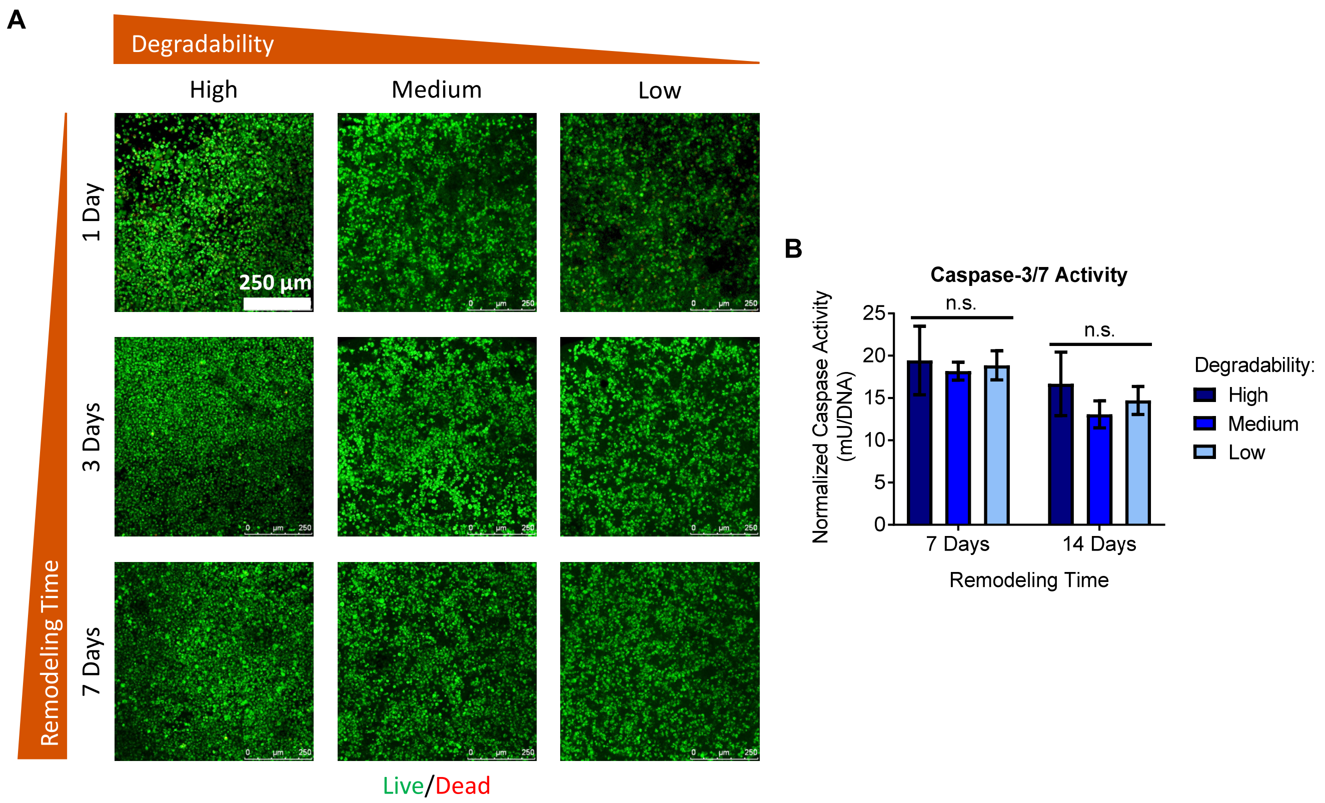

Supplemental Figure S3. NPCs remain highly viable in all hydrogel formulations. **(A)** Encapsulated NPCs exhibit high membrane integrity and maintain enzymatic activity, as visualized by live/dead staining. **(B)** No difference in caspase-3/7 activity is observed across hydrogel formulations after 7 or 14 days of culture, indicating no significant differences in apoptotic cell death among hydrogel conditions. Data are mean ± s.d. n.s. = not significant ($p>0.05$), two-way ANOVA with Bonferroni post-hoc test, n=3-4.

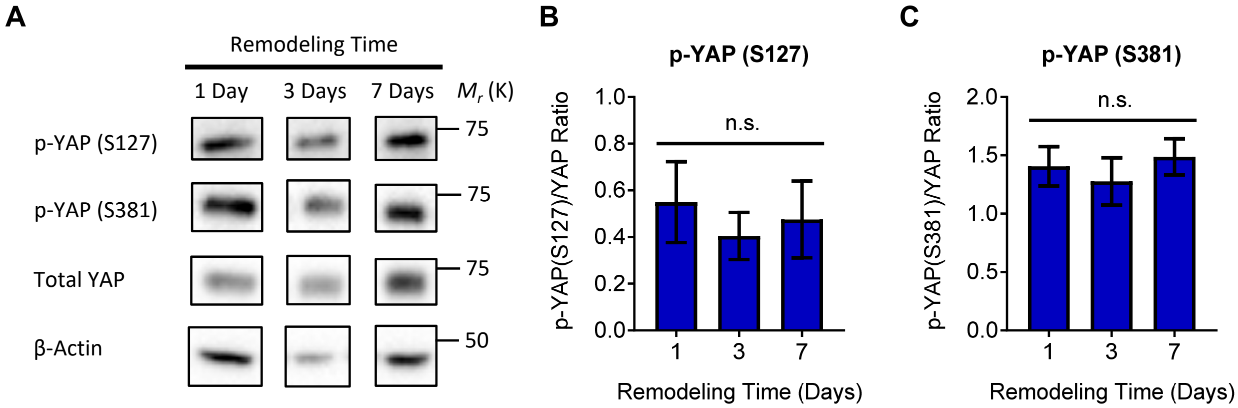

Supplemental Figure S4. YAP phosphorylation does not vary as a function of remodeling time. **(A)** Representative Western blots for phospho-YAP (S127), phospho-YAP (S381), and total YAP for NPCs encapsulated in high degradability hydrogels and provided different amounts of remodeling time. β-actin was used as a loading control. Quantification of Western blots for **(B)** phospho-YAP (S127) and **(C)** phospho-YAP (S381) revealed no significant differences in the ratios of phosphorylated to total YAP at any time point tested. Data are mean ± s.e.m. n.s. = not significant ($p>0.05$), one-way ANOVA with Bonferroni post-hoc test, n=4.

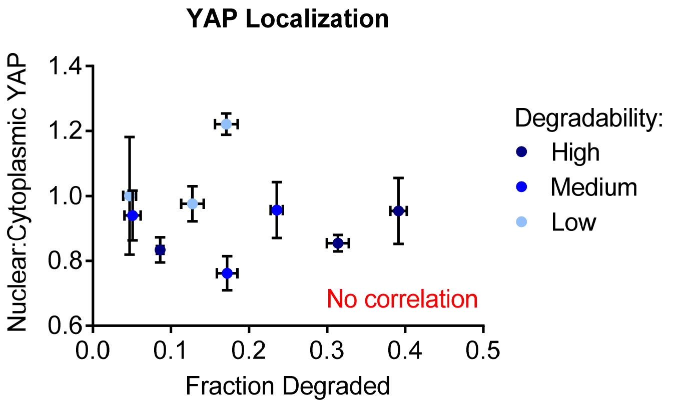

Supplemental Figure S5. YAP localization is not correlated with extent of hydrogel degradation in any hydrogel formulation tested. The ratio of nuclear:cytoplasmic YAP was calculated for NPCs encapsulated in hydrogels of varying degradability at 1, 3, and 7 days post-encapsulation. The fraction of hydrogel degraded for each condition at each time point was obtained from the data in Figure S1B. YAP data are mean ± s.d. Degradation data are plateau degradation values ± s.e. Spearman correlation ($p=0.58$), $n=3$.

Supplemental Video S1. Time lapse imaging of intracellular calcium concentration after treatment with GABA. The numbered cells correspond to those identified in Figure 4A.

Supplemental Video S2. Time lapse imaging of intracellular calcium concentration after treatment with glutamate. The numbered cells correspond to those identified in Figure 4B.

Supplemental Video S3. Time lapse imaging of intracellular calcium concentration without neurotransmitter treatment. Few spontaneous changes in intracellular calcium concentration were observed.

Supplemental Table 1. Characterization of ELP hydrogels.

**Crosslinker: Protein Reactive Groups**	**Relative Degradability**	**Elastic Modulus [*E*, Pa] (mean±s.d.)**	**Maximum Fraction Degraded (plateau±s.e.)**	**Degradation Rate [hr^{-1}] (*k*±s.e.)**
0.5:1	High	590±60	0.42±0.008	0.014±0.0005
0.75:1	Medium	960±140	0.27±0.005	0.013±0.0008
1:1	Low	1450±240	0.19±0.004	0.012±.0.0008

Supplemental Table S2. Primers used in qRT-PCR.

**Target**	**Primers**
Nestin	Fwd: CCCTGAAGTCGAGGAGCTG<br>Rev: CTGCTGCACCTCTAAGCGA
Sox2	Fwd: GCGGAGTGGAAACTTTTGTCC<br>Rev: CGGGAAGCGTGTACTTATCCTT
VGLUT1	Fwd: GGTGGAGGGGGTCACATAC<br>Rev: AGATCCCGAAGCTGCCATAGA
MASH1	Fwd: GCAACCGGGTCAAGTTGGT<br>Rev: GTCGTTGGAGTAGTTGGGGG
β-tubulin III	Fwd: TAGACCCCAGCGGCAACTAT<br>Rev: GTTCCAGGTTCCAAGTCCACC
MAP2	Fwd: GCCAGCCTCAGAACAAACAG<br>Rev: AAGGTCTTGGGAGGGAAGAAC
MAPτ	Fwd: CGCTGGGCATGTGACTCAA<br>Rev: TTTCTTCTCGTCATTTCCTGTCC
GFAP	Fwd: CGGAGACGCATCACCTCTG<br>Rev: AGGGAGTGGAGGAGTCATTCG
S100β	Fwd: TGGTTGCCCTCATTGATGTCT<br>Rev: CCCATCCCCATCTTCGTCC
Olig2	Fwd: TCCCCAGAACCCGATGATCTT<br>Rev: CGTGGACGAGGACACAGTC
CNPase	Fwd: TTTACCCGCAAAAGCCACACA<br>Rev: CACCGTGTCCTCATCTTGAAG
YAP	Fwd: TACTGATGCAGGTACTGCGG<br>Rev: TCAGGGATCTCAAAGGAGGAC

Supplemental Table S3. Primary antibodies.
ICC = Immunocytochemistry. WB = Western blot.

Target	Host Species	Supplier	Catalog Number	Dilution
Nestin	Mouse	BD Pharmingen	556309	WB: 1:1000
Sox2	Rabbit	Millipore	AB5603	WB: 1:2000
GFAP	Chicken	Aves Labs	GFAP	ICC: 1:300
S100β	Rabbit	Abcam	ab52642	ICC: 1:400
β-tubulin III	Chicken	Aves Labs	TUJ	ICC: 1:1000
MAP2	Rabbit	Millipore	AB5622	ICC: 1:400
Doublecortin	Goat	Santa Cruz Biotechnology	sc-8066	ICC: 1:400
Neurofilament	Mouse	BioLegend	837904	ICC: 1:400
YAP	Rabbit	Cell Signaling Technology	14074S	ICC: 1:400<br>WB: 1:1000
p-YAP (S127)	Rabbit	Cell Signaling Technology	13008S	WB: 1:1000
p-YAP (S381)	Rabbit	Cell Signaling Technology	13619S	WB: 1:1000
β-actin	Rabbit	Cell Signaling Technology	4970S	WB: 1:1000

Supplemental Table S4. shRNA sequences.

shRNA	Mature Antisense Sequence
Non-silencing	CTTACTCTCGCCCAAGCGAGAG
ADAM9	TTGGTAACACCTCAACTGT
